# Responding to reports of nitazene toxicity in Australia

**DOI:** 10.5694/mja2.52605

**Published:** 2025-02-12

**Authors:** Brendan Clifford, Amy Peacock, Krista J Siefried, John Gobeil, Jennifer L Smith, Nadine Ezard

**Affiliations:** ^1^ National Centre for Clinical Research on Emerging Drugs University of New South Wales Sydney NSW; ^2^ St Vincent's Hospital Sydney Sydney NSW; ^3^ National Drug and Alcohol Research Centre University of New South Wales Sydney NSW; ^4^ Australian Injecting and Illicit Drug Users League Canberra ACT; ^5^ University of Western Australia Perth WA; ^6^ Centre for Clinical Research in Emergency Medicine Royal Perth Hospital Perth WA

**Keywords:** Overdose, Disasters, Toxicology

Nitazenes are a class of synthetic 2‐benyzl‐benzimidazole opioid receptor agonists that can be several hundred times the potency of morphine.^1^ Clonitazene and etonitazene were initially developed in the 1950s as potential analgesics, but were never approved for therapeutic purposes due to their high potency.^1^ Isotonitazene was first notified as a drug of concern to the United Nations Office on Drugs and Crime (UNODC) in 2019. By the beginning of 2024, 13 different nitazene analogues across six global regions had been reported, highlighting the potential for continued proliferation of chemically modified structures into the drug market.^2^ Nitazene‐associated harms and deaths have been reported in North America and Europe, with toxicity symptoms being similar to those from other opioids, including respiratory depression and decreased level of consciousness.^1^

## Nitazenes in Australia

Nitazenes are now an established feature of the Australian illicit drug market,^3^ with 11 nitazenes classified as Schedule 9 (prohibited substance) by the Therapeutic Goods Administration.^4^ The first confirmed nitazene detections in Australia were in 2021, including reports in New South Wales^5^ and Victoria.^6^ Sentinel toxico‐surveillance of Australian and Victorian emergency department presentations analytically confirmed the protonitazene, metonitazene, isotonitazene, butonitazene, etodesnitazene and etonitazepyne across 32 cases between July 2020 and February 2024.^7^ Wastewater analysis has not detected nitazenes in Australia, but routine monitoring by this method for new psychoactive substances is limited.^8^ A recent retrospective study of national coronial data identified 17 deaths due to nitazene (etodesnitazene, metonitazene and protonitazene) toxicity, with the first in 2021.^9^ Nitazenes have also been identified in police and customs seizures in multiple jurisdictions, including the Northern Territory and NSW.^3^ Analysis of cryptomarkets has indicated an increase in availability of nitazenes for sale online in Australia between February 2023 and January 2024.^10^

Government‐issued public drug alerts are intended to raise awareness of new risks in the drug market and are issued by health departments in some, but not all, Australian jurisdictions. A list of alerts related to nitazenes is provided in the Box, with the relevant harms reported in the alert, where available. Criteria for the issuing of drug alerts vary between jurisdictions, so the box is indicative of the types of harms that may be caused by nitazenes, rather than a comprehensive list. Public drug alerts related to nitazenes have been issued in the Australian Capital Territory,^11^ NSW,^12^ Queensland,^13^ SA^11^ and Victoria.^14^ Nitazenes have been found to be represented as or in other opioids such as heroin, and several non‐opioid substances: gamma‐hydroxybutyrate (GHB), ketamine, cocaine, 3,4‐methylenedioxymethamphetamine (MDMA) and the mescaline analogue, 3,5‐dimethoxy‐4‐propoxyamphetamine (3C‐P).^3^ Nitazenes have also been presented as counterfeit forms of pharmaceutical oxycodone and alprazolam. Routes of administration for nitazenes are similarly varied, with oral, insufflation, vaporised, rectal and injection routes reported.^3^

Box 1 **Nitazene drug alerts issued by Australian states and territories**

**Table:** 

Date	Nitazene	State or territory	Sold as	Harms reported
Nov 2024	Isotonitazepyne	NSW^12^	Oxycodone	Two hospitalisations
Nov 2024	Isotonitazepyne	Queensland^11^	Oxycodone	Detection only
July 2024	Protonitazene	SA^11^	Oxycodone	Overdose
July 2024	Protonitazene	Victoria^14^	Cocaine	Serious harms
May 2024	Protonitazepyne Protonitazene	NSW^12^	Etazene, cocaine or ketamine	Four hospitalisations
Apr 2024	Nitazene	NSW^12^	Heroin	Opioid overdoses
Mar 2024	Protonitazene	Victoria^14^	3‐CP	One hospitalisation
Jan 2024	Nitazene	NSW^12^	MDMA	Multiple hospitalisations
Nov 2023	Protonitazene	Queensland^13^	Benzodiazepines	Detection only
Oct 2023	Nitazene	SA^15^	Not reported	Detection only
Aug 2023	Metonitazene	Victoria^14^	Cocaine	Two hospitalisations
July 2023	Protonitazene	SA^11^	GHB, methamphetamine	Two overdoses
May 2023	Isotonitazene	NSW^12^	Yellow powder	Serious harms, including possible deaths
Dec 2022	Nitazene	NSW^12^	Heroin	Increased hospitalisations and ICU admissions
Dec 2022	Metonitazene	ACT^11^	Oxycodone	Detection only
June 2022	Protonitazene	Victoria^14^	Ketamine	Serious recent hospitalisations
Aug 2022	Etodesnitazene	NSW^12^	Alprazolam	Detection only

3‐CP = 3,5‐dimethoxy‐4‐propoxyamphetamine; ACT = Australian Capital Territory; GHB = gamma hydroxybutyrate; ICU = intensive care unit; MDMA = 3,4‐methylenedioxymethamphetamine; NSW = New South Wales; SA = South Australia.

There is concern that synthetic opioid production may increase due to recent political changes in Afghanistan, a key producer of heroin, which have resulted in a marked decrease in global opium supply.^15^ While the full effects of this disruption to the illicit drug market are not immediately apparent, it is important to consider preparedness in Australia for changes in drug use, including increases in intended or unintentional exposure to synthetic opioids such as nitazenes.

## Detection and monitoring of nitazenes

Responding promptly to nitazenes and other emerging drugs is dependent on early recognition of their presence in the community. Given the nature of illicit drug use, public health monitoring relies on multiple data sources to understand the presence, use and related harms of emerging drugs. In addition to traditional monitoring indicators, such as drug use surveys and hospital presentation data, methods that provide toxicological verification (eg, emergency department surveillance, drug checking, syringe residue, wastewater monitoring, police seizure testing), as well as more informal data sources (eg, anecdotal reports from people who use drugs and frontline service providers, online data) are necessary to provide early signals of use in the community and broader shifts over time. Prompt responses, such as issuing drug alerts, depend on systems for knowledge exchange, decision‐making processes, and information dissemination across multiple stakeholder groups (including peer‐led drug user organisations [DUOs] and non‐health sectors such as police) both within and across jurisdictions.

## Responding to nitazenes

Drug alerts, including drug notifications issued by community organisations, play an important role in ensuring an informed community and health response. In an Australian study of engagement with drug alerts, over half of the survey participants (567 people who used illicit drugs) changed their use of the drug mentioned in the alert, either by stopping use of the drug type entirely (18%), avoiding using the specific drug matching the alert (20%) or changing their use behaviours (18%).^16^ There are a number of considerations to optimise the benefits of drug alerts, such as how to provide information that is both concise, but also relevant for a range of knowledge levels.^17^ Meaningful and appropriately resourced collaboration with people with lived and living experience of drug use is essential to ensure not just effective monitoring, but also responses to emerging drugs of concern. The role of peer‐led DUOs in facilitating these partnerships cannot be understated. Incorporation of lived and living experience into risk communication design enhances the uptake of messaging by ensuring acceptability and relevance of language and dissemination methods.

The opioid antagonist naloxone is effective at reversing nitazene toxicity,^7^ although higher and repeated doses of naloxone may be needed given the higher potency and potential longer duration of nitazenes compared with more commonly used opioids.^18^ All pharmacies in Australia can supply naloxone for free in the community through the Commonwealth‐funded Take Home Naloxone Program, although there is room to increase the number of pharmacies participating, and to support pharmacists in the provision of naloxone education.^19^ People who use opioids and who are aware of naloxone may not be familiar with the possible need for higher and repeated doses. Due to the variety and unpredictability of nitazenes, emergency medical services should be called when an opioid overdose is suspected. Importantly, the presence of nitazenes in non‐opioid substances underscores the need to broaden naloxone distribution to populations (such as people who use methamphetamine or other stimulants) and settings (such as music events) not traditionally associated with opioid use. In addition to distribution by clinical and harm reduction services, carriage of naloxone by police has been found in an evaluation in Western Australia to be feasible, effective and may save lives through enabling prompt response to overdose^20^ as well as demonstrating a commitment to protect the wellbeing of all in the community.

In addition to naloxone distribution, harm reduction services, such as needle syringe programs and supervised consumption rooms, empower people to reduce the risk of harm. Such services are an important trusted source of information^21^ and play a central role in community‐led education efforts given the relatively low awareness of nitazenes even in populations with experience in opioid use and harm reduction. Harm reduction messaging for nitazenes is similar to that for other opioids, including avoiding using when alone or mixing with other drugs. Staggering use when using as a group is also recommended due to the potential for a rapid onset of loss of consciousness.

Immunoassay test strips have been used as a low cost harm reduction tool to allow people who use drugs to test for the presence of certain substances (eg, fentanyl), and the effectiveness of strips recently developed for nitazenes is being established.^22^ The ACT, Queensland, New South Wales and Victoria have drug checking services either in operation or in the planning stage. Drug checking services also afford an opportunity for education on health and harm reduction and appear to be effective at influencing the behaviours of people who use drugs.^23^

Sufficient access to opioid dependence treatment (ODT) programs, including the expanding range of ODT medications, reduces the risk of overdose, bloodborne virus transmission and all‐cause mortality.^24^ Although ODT medications have been recently included in the Pharmaceutical Benefits Scheme, thereby increasing their affordability, there is considerable concern around the current capacity to meet ODT need given the reducing number of available prescribers and dispensaries. Preparedness for the increasing use of potent opioids, such as nitazenes, necessitates urgent updating of national guidelines for medication‐assisted ODT^25^ to account for developments in long‐acting formulations (such as injectable buprenorphine) and expanded roles for nurse practitioner prescribing, pharmacy dispensing and administration.

## Conclusion

The extent to which nitazenes will become a feature of the Australian drug use landscape is unknown, but there are sufficient signals to consider preparedness for an increase in opioid‐related harms. People with lived and living experience of drug use must be central to preparedness planning and responses. Effective monitoring of drug use patterns and a resourced public health and harm reduction sector not only provides some readiness for nitazenes, but also for other drugs of concern as they emerge.

## Open access

Open access publishing facilitated by University of New South Wales, as part of the Wiley – University of New South Wales agreement via the Council of Australian University Librarians.

## Competing interests

Amy Peacock has received untied educational grant funding from Seqirus and Mundipharma for post‐marketing surveillance of opioid medications in Australia.

## Provenance

Not commissioned; externally peer reviewed.
